# Statewide mental health training for probation officers: improving knowledge and decreasing stigma

**DOI:** 10.1186/s40352-017-0057-y

**Published:** 2017-11-15

**Authors:** Nikhil Tomar, Marilyn A. Ghezzi, Lauren Brinkley-Rubinstein, Amy Blank Wilson, Tonya B. Van Deinse, Stacey Burgin, Gary S. Cuddeback

**Affiliations:** 1 Division of Occupational Science and Occupational Therapy, University of North Carolina at Chapel Hill, 321 South Columbia Street, CB#7122, Chapel Hill, NC 27599 USA; 2 School of Social Work, University of North Carolina at Chapel Hill, 325 Pittsboro Street, CB#3550, Chapel Hill, NC 27599 USA; 3 School of Medicine, Department of Social Medicine and Center for Health Equity Research, University of North Carolina at Chapel Hill, 333 South Columbia Street, CB#7240, Chapel Hill, NC 27599 USA; 4 School of Medicine, Department of Psychiatry, Center for Excellence in Community Mental Health, University of North Carolina at Chapel Hill, 200 N. Greensboro St., Suite C-6, Carrboro, NC 27510 USA

**Keywords:** Stigma, Mental health knowledge, Probation officers, Survey

## Abstract

**Background:**

The large and growing number of probationers with mental illnesses pose significant challenges to the probationer officers who supervise them. Stigma towards mental illnesses among probation officers is largely unstudied and the effectiveness of training initiatives designed to educate probation officers about mental illness is unknown. To address these gaps in the literature, we report findings from a statewide mental health training initiative designed to improve probation officers’ knowledge of mental illnesses. A single-group pretest posttest design was used and data about stigma towards mental illnesses and knowledge of mental illnesses were collected from 316 probation officers. Data were collected prior to and shortly after officers viewed a series of educational training modules about mental illnesses.

**Results:**

Officers’ knowledge of mental illnesses increased and officers demonstrated lower levels of stigma towards persons with mental illnesses as evidenced by scores on a standardized scale.

**Conclusion:**

Mental health education can help decrease stigma and increase knowledge of mental illnesses among probation officers. More research is needed to assess the impact of these trainings on probationers’ mental health and criminal justice outcomes.

## Background

In the United States, there were approximately 3.86 million adults on probation at 2014 yearend (Kaeble et al. 2015). Best estimates suggest that 16–27% of adults on probation have a mental illness, which is of concern given evidence that probationers with mental illnesses are more likely to experience revocations and are more likely to be re-arrested compared to probationers without mental illnesses (Kaeble et al. 2015; Crilly et al. 2009; Ditton 1999; Cloyes et al. 2010; Ostermann and Matejkowski 2012). The number of probationers with mental illnesses is large and growing and community corrections has become, to some extent, a de-facto mental healthcare system for many (Kaeble et al. 2015; Crilly et al. 2009; Ditton 1999; Cloyes et al. 2010; Ostermann and Matejkowski 2012; Lamb et al. 2004), which often places significant strain on traditional probation officers (Skeem et al. 2006). For example, due to lack of training and knowledge of mental illness, probation officers may perceive probationers with mental illnesses as high-risk offenders who require more surveillance than those without such diagnoses, which increases the chance for noticing minor infractions that results in probation revocations and re-incarcerations (Eno Louden et al. 2008; Eno Louden and Skeem 2013; Porporino and Motiuk 1995; Petersilia and Turner 1993).

Indeed, there is evidence that revocation and recidivism among probationers with mental illnesses is associated with knowledge about and stigma towards mental illnesses among probation officers (Eno Louden et al. 2008; Eno Louden and Skeem 2013). Stigma is a process where a socially undesirable label (e.g., a psychiatric patient, mentally ill) is attached with negative stereotypes leading to prejudice and discrimination (Link and Phelan 2001). Stigma is closely associated with knowledge about an illness and stigma about mental health can be compounded based on other stigmatizing characteristics (e.g. race and gender) or life experiences, including involvement in the criminal justice system (Link and Phelan 2001; Gonzalez et al. 2005). To further compound matters, stigma may also vary by diagnosis in that evidence suggests there is more public stigma towards schizophrenia than towards depression (Wood et al. 2014; Pescosolido et al. 2010).

Stigma and mental health knowledge are of special concern in the context of probation as decisions about risk assessment and management are often made on the basis of probationer-officer interactions, which can be influenced by officers’ knowledge about and stigma towards mental illnesses (Eno Louden et al. 2008; Eno Louden and Skeem 2013). For example, traditional probation officers are more likely to endorse punitive strategies (e.g., issuing a violation) for non-compliance as compared to specialty mental health probation officers who have extensive training in mental health education and are more likely to employ problem-solving strategies with probationers (Eno Louden et al. 2008). Although officers’ lack of knowledge about mental health and stigma towards mental illnesses can contribute to undesirable outcomes for probationers with mental illnesses, there is also evidence that mental health knowledge can help reduce stigma among probation officers and improve probationers’ criminal justice outcomes (Eno Louden et al. 2008; Link and Phelan 2001; Pinfold et al. 2003).

Evidence suggests that education and training can reduce mental illness stigma among police officers and improve behavioral outcomes such as communicating with and willingness to work with an individual with mental illness (Pinfold et al. 2003; Hansson and Markström 2014); however, there is limited information about improving mental health knowledge and reducing stigma related to mental illnesses among probation officers. More research is needed to explore the role of mental health education and its impact on stigma among probation officers and feasible and practical strategies to deliver mental health education to community corrections. To address these gaps in the literature, we report findings from a statewide mental health training initiative among probation officers in a large Southeastern state.

## Method

### Design

A single-group pretest-posttest design was used to evaluate the effects of a mental health training program on stigma towards mental illnesses and knowledge of mental illnesses among probation officers in a large Southeastern state. Per the requirements of the state’s Department of Public Safety, all probation officers were mandated to view six mental health training modules (see below) as a part of a statewide effort to increase officers’ awareness of mental illnesses. Prior to viewing the modules, 1691 officers were invited to complete a pretest survey, which included items about stigma toward mental illnesses and mental health knowledge. Four weeks after the deadline to complete the modules, officers were invited to complete a posttest. Probation officers were recruited through email and completed both pretest and posttest measures online. Data collection occurred from October 2014 through February 2015. This study was approved by the Institutional Review Board at the (omitted to preserve authors’ anonymity).

### Sample

Survey invitations were emailed to 1691 probation officers across the state; however, due to technical difficulties involving email security and firewall issues, only 582 officers received and competed the pretest survey. A total of 368 officers completed both the pretest and the posttest survey but 52 surveys were unusable due to missing data, resulting in an analytic sample of 316 probation officers (19% of all officers across the state). The analytic sample of 316 was 71% (*n* = 223) white, 26% (*n* = 81) African American and was mostly female (53%, *n* = 166) with 47% (*n* = 150) males. The average age of respondents was 42.18 (SD = 9.4) with a range of 23–63 years. Also, 80% (*n* = 254) of the sample had a college degree, 6% (*n* = 19) had a graduate degree, and 50% (*n* = 158) worked in rural settings.

### Measures

A standardized 11-item measure of stigma originally developed for inclusion in the 2006 HealthyStyles survey was used (Kobau et al. 2010). The measure uses a 5-point Likert-type response pattern to indicate level of agreement, where 1 = strongly disagree and 5 = strongly agree. Higher scores on the measure indicate less stigma toward mental illnesses. The measure has acceptable internal consistency (Cronbach’s alpha ranging between 0.66–0.69) and convergent validity (reliability coefficient ranging between 0.69–0.70) (Hansson and Markström 2014).

Knowledge of mental illnesses was assessed using a 15-item researcher-developed measure. For example, one of the items asked, “Which of the following is a symptom of schizophrenia? Choose the best answer.” Another item asked, “One of the offenders on your caseload is experiencing a crisis. What would be a good way to respond? Choose the best answer.” This measure used a multiple-choice response format. Scores on the measure ranged from 0 to 15 with higher scores indicating greater mental health knowledge.

### Statewide mental health training

The statewide mental health training program, part of a larger study regarding specialty mental health probation, was developed to increase probation officers’ knowledge and awareness of mental illnesses. The training modules, which, at the request of a state’s Department of Public Safety (DPS), were intended to be introductory and general in their nature. In particular, DPS requested that the training provide an overview of severe and persistent mental illnesses, and provide information about key diagnoses and the medications and services associated with those diagnoses. The training consisted of six modules delivered as narrated PowerPoint presentations. The modules were developed after reviewing curricula that provide information about basic mental health issues for the lay public, such as Mental Health First Aid, Crisis Intervention Training, and other sources, such as Mental Health America and the National Institute of Mental Health. Authors’ own materials used to teach graduate students were also incorporated into the modules. The final versions of the training modules were informed by feedback from key stakeholders at the Department of Public Safety and by four specialty mental health probation officers. The six modules are as follows:

1.     
*Determining the need for a mental health referral*: This module is intended to help probation officers interpret the results of a mental health scale, which is completed by all probationers. The module includes suggestions for follow up questions to help officers and probationers have a discussion about mental health, how to interpret the answers to the mental health questions and what steps to take regarding referrals to mental health providers.
2.     
*Severe and Persistent Mental Illness (SPMI)*: Specifically, this module defines severe and persistent mental illness, explains major mental disorders, including schizophrenia, bipolar disorder and major depression, and addresses co-occurring mental illness and substance use.
3.     
*Medications*: In this module, major medications are presented as well as possible reasons for medication non-adherence. Also, suggestions for officers with regard to having conversations with probationers about medications are presented.
4.     
*Other mental health disorders*: In this module, personality disorders, especially borderline and antisocial personality disorders, as well as post-traumatic stress disorder (PTSD) and traumatic brain injury (TBI) are explained.
5.     
*Responding to Crisis*: This module explains mental health crises, how to tell if a probationer is in crisis, and how to communicate with a probationer who is in crisis. Procedures for making referrals for a mental health crisis are described and additional resources are provided.
6.     
*Self-care for Probation Officers:* This module discusses burnout and vicarious traumatization that probation officers may experience. It describes how to recognize symptoms of stress and burnout and provides additional resources.

### Data analysis

Paired sample t-tests were conducted to examine differences in pre- and posttest scores on the measures of stigma and mental health knowledge. Next, chi-square tests, t-tests and Analysis of Variance (ANOVA) were conducted to examine relationships between stigma and mental health knowledge, and differences in scores based on various demographic characteristics. All the analyses were conducted using SAS V9.3.

## Results

The average score at pretest on the stigma measure was 37.14 (SD = 4.7) and the average score at posttest was 37.94 (SD = 4.77) and this difference was statistically significant (t(315) = −3.88, *p* < .001). The average score on the mental health knowledge measure at pretest was 11.72 (SD = 1.94) and the average score at posttest was 12.17 (SD = 2.39), and this was a statistically significant difference (t(315) = −3.05, *p* < .01).

Compared to pretest scores, posttest scores on the stigma measurement significantly decreased for both male (t(149) = −1.96, *p* = .05) and female (t(165) = −3.60, *p* < .001) officers. Further, female officers scored significantly higher on stigma measure (indicating less stigma towards persons with mental illnesses) in both pre- (t(295.69) = 3.57, *p* < .001) and posttest surveys (t(303.04) = 4.18, *p* < .001). Pre- and posttest scores on the mental health knowledge scale did not differ significantly between genders. Scores on either measure did not differ significantly on the basis of race or ethnicity, age or education status. Also, no significant difference between urban and rural county officers’ scores on either measure was found.

## Discussion

The major finding for this study is that mental health education training has the potential to increase mental health knowledge and decrease stigma towards mental illnesses among probation officers. The mental health modules provide a feasible and effective strategy to increase knowledge and reduce stigma related to mental illnesses and this is important for local and state criminal justice authorities that have limited training resources and time. The findings presented here can be used to support the implementation of similar trainings in probation settings where in-person trainings are not a viable option. Although training is likely a necessary but not sufficient solution to reducing recidivism among probationers with mental illnesses; web-based learning modules could be a reasonable starting point (on a practice level) in providing probation officers the knowledge and information needed to assist probationers with mental illnesses. Future research is needed to assess the impact of such trainings on probationer outcomes, such as revocations and recidivism.

While the scores on measures of mental health knowledge and stigma did not vary on most demographic characteristics, there were statistically significant differences between male and female officers. Female officers demonstrated less stigma towards persons with mental illnesses in both pre- and posttest measures. This finding is aligned with previous research suggesting that females generally report less negative attitudes regarding mental illnesses than males (Gonzalez et al. 2005). However, this difference cannot be attributed to differences in mental health knowledge as no such difference between male and female officers was found in this study.

### Limitations

A limitation of this study is the inability to account for social desirability, a concern for survey research pertaining to sensitive topics, such as mental illness. No items to detect social desirability (i.e., responding to survey items in a way that respondents think will be favored by others) were included in the survey (Tourangeau et al. 2000). However, the surveys were collected anonymously, which likely reduced the potential for social desirability among respondents (Tourangeau et al. 2000). Another limitation is that the mental health knowledge questionnaire employed in this study is not a standardized measure and has not been evaluated for its psychometric properties. The study design did not incorporate a comparison group of officers who did not receive mental health training to compare levels of stigma or mental health knowledge. Thus, alternative explanations for decreased stigma and increased mental health knowledge among officers in our sample cannot be ruled out. However, it is noteworthy that while the probation officers demonstrated relatively high baseline mental health knowledge, there were still improvements on the post-training scores.

Links to the survey were administered via officers’ state employee email addresses, which potentially were blocked by the state’s email security and firewall protocols. Thus, another limitation is the low response rate, which is often a challenge irrespective of mode of survey administration (Tourangeau et al. 2000; Dillman 2011). Testing survey distribution methods prior to dissemination to the full sample is always good practice. Although technical difficulties during the administration of the survey presented challenges to data collection and led to a smaller sample of officers, it is noteworthy that the response rate was 63% (368/582) among those officers who received emails and survey links.

The extent to which the responses of the sample can be generalized to other probation settings is unclear, however, and caution is warranted in generalizing these findings more broadly. Also, the long-term influence of the mental health training modules on stigma could not be determined as no follow-up surveys were administered to observe changes in officers’ interactions with offenders with mental illnesses.

## Conclusion

Stigma is a significant aspect of the lived experience of persons with mental illnesses (Link and Phelan 2001). It is important to address stigma among probation officers regarding mental illnesses and, consequently, provide tools to address the influence of stigma on officer-probationer interactions. This study found that mental health education modules can be effective in reducing stigma. However, more research is required to assess direct or indirect influence of such trainings on recidivism, hospitalization, and community reintegration of probationers and the long-term effect on probation officers’ attitudes and actions. Future research should also assess the feasibility and effectiveness between delivery methods, such as online and face-to-face format, along with long-term influence of such trainings.
